# Can we identify individuals with an *ALPL* variant in adults with persistent hypophosphatasaemia?

**DOI:** 10.1186/s13023-020-1315-y

**Published:** 2020-02-17

**Authors:** C. Tornero, V. Navarro-Compán, J. A. Tenorio, S. García-Carazo, A. Buño, I. Monjo, C. Plasencia-Rodriguez, J. M. Iturzaeta, P. Lapunzina, K. E. Heath, A. Balsa, P. Aguado

**Affiliations:** 1 Department of Rheumatology, La Paz University Hospital, IdiPAZ, Paseo de la Castellana, 261, 28046 Madrid, Spain; 2 Institute of Medical and Molecular Genetics (INGEMM), La Paz University Hospital, IdiPAZ, Universidad Autónoma de Madrid, Madrid, Spain; 3 CIBERER (Centro de Investigación Biomédica en Red de Enfermedades Raras), ISCIII, Madrid, Spain; 4 Department of Clinical Biochemistry, La Paz University Hospital, Madrid, Spain; 5 Skeletal dysplasia multidisciplinary Unit (UMDE), La Paz University Hospital, Madrid, Spain

**Keywords:** Metabolic bone diseases, Hypophosphatasia, Hypophosphatasaemia, Alkaline phosphatase, *ALPL*

## Abstract

**Background:**

Hypophosphatasia (HPP) is an inborn error of metabolism characterized by low levels of serum alkaline phosphatase (ALP). Scarce evidence exists about features that should signal the potential association between hypophosphatasaemia and HPP in adults. The aim of this study is to estimate the prevalence of *ALPL* variants in subjects with persistent hypophosphatasaemia and determine the associated clinical and laboratory features. For this cross-sectional study, laboratory records of 386,353 subjects were screened by measurement of ALP activity. A total of 85 (0.18%) subjects with persistent hypophosphatasaemia (≥2 serum alkaline phosphatase–ALP–measurements ≤35 IU/L and none > 45 IU/L) were included (secondary causes previously discarded). *ALPL* genetic testing and a systematized questionnaire to retrieve demographic, clinical and laboratory data were performed. Descriptive analysis and logistic regression models were employed to identify the clinical and laboratory characteristics associated with *ALPL* variants.

**Results:**

Forty subjects **(**47%) had a variant(s) in *ALPL*. With regard to clinical characteristics, the presence of an *ALPL* variant was significantly associated only with musculoskeletal pain (OR: 7.6; 95% IC: 1.9–30.9). Nevertheless, a trend to present more dental abnormalities (OR: 3.6; 95% IC: 0.9–13.4) was observed. Metatarsal stress fractures were also more frequent (4 vs 0; *p* < 0.05) in this group. Regarding laboratory features, median ALP levels were lower in subjects with *ALPL* variants (26 vs 29 IU/L; *p* < 0.005). Interestingly, the threshold of ALP levels < 25 IU/L showed a specificity, positive predictive value and positive likelihood ratio of 97.8, 94.4% and 19.8 to detect a positive *ALPL* test, respectively.

**Conclusions:**

In subjects with persistent hypophosphatasaemia –secondary causes excluded– one out of two presented *ALPL* variants. Musculoskeletal pain and ALP levels < 25 IU/L are associated with this variant(s). In this scenario, ALP levels < 25 IU/L seem to be very useful to identify individuals with the presence of an *ALPL* variant.

## Background

Hypophosphatasia (HPP) is an inborn error of metabolism characterized by low levels of serum alkaline phosphatase (ALP) caused by loss-of-function variants. This results in variants in *ALPL*, located on chromosome 1p36.1-p34, which encodes the tissue-nonspecific alkaline phosphatase [1, 2]. Impaired ALP activity can lead to the extracellular accumulation of ALP-specific substrates, such as inorganic pyrophosphate (PPi), a potent inhibitor of bone and dental mineralization [3].

HPP is a rare disease with an estimated prevalence in Europe of 1/300,000 in severe cases and of 1/6370 in moderate cases [4]. HPP covers a wide spectrum of clinical manifestations whose severity inversely correlates with the age of onset [5, 6]. Clinical features range from lethal phenotypes or rickets in early ages to musculoskeletal pain, chondrocalcinosis, calcific periarthritis or fractures in adults [5, 7].

The genetic background of subjects may impact the clinical course of HPP: recessive inherited variants are commonly associated with more severe manifestations, while both autosomal dominant and recessive inheritance may occur in milder forms [8]. Hence, efforts have been made to identify *ALPL* disease-causing variants and their pattern of inheritance. To date, more than 380 variants have been described [9]. In addition, a high phenotypic variability within members of the same family sharing the same disease causing variants has been observed [8, 10].

The main biochemical hallmark of HPP is hypophosphatasaemia; however, in clinical practice, low ALP levels are often overlooked and their causes are not usually investigated [6, 11], even when evaluating bone fragility. This lack of clinical attention often leads to erroneous diagnoses and prescription of contraindicated drugs in HPP, such as antiresorptive agents [12, 13].

Scarce evidence exists about features that should signal the potential association between hypophosphatasaemia and HPP in adults. Previous studies have shown a prolonged delay in the diagnosis of HPP [5, 14, 15] and access to the genetic testing is not always possible or rapid testing may be required.

In this context, the aims of this study were to estimate the prevalence of subjects with variants in *ALPL* among those with persistent hypophosphatasaemia and to determine the clinical and laboratory features associated with the presence of *ALPL* variants and their utility to identify a positive genetic test in the diagnostic work-up of HPP.

## Results

### Study population

**Fig. 1:**
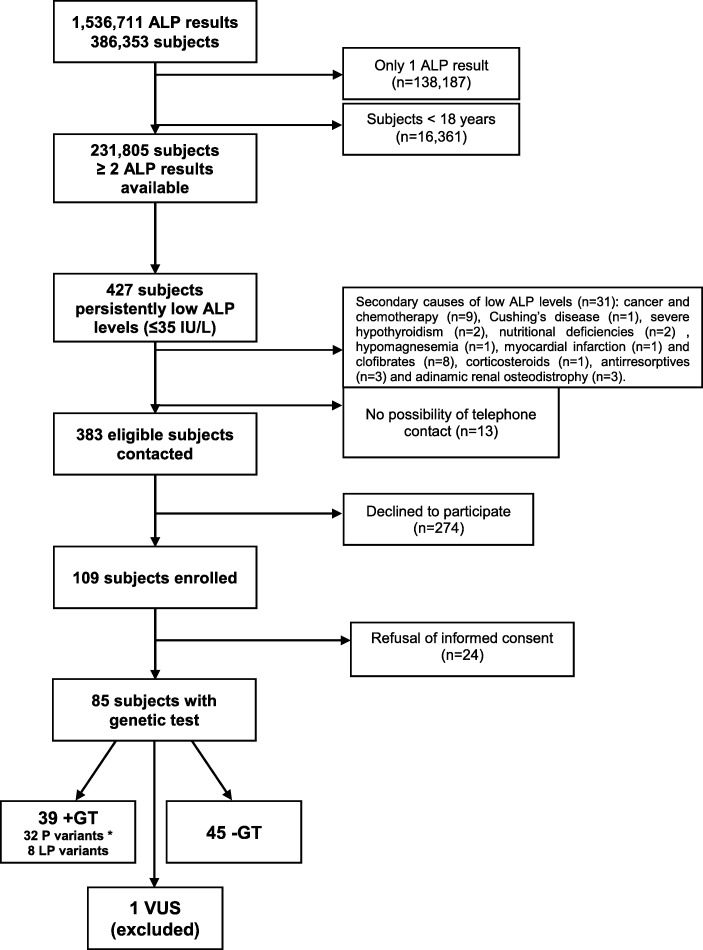
Flowchart describing screening and selection process. ALP = alkaline phosphatase; +GT and –GT refer to subjects with either presence or absence of disease causing variants in *ALPL*, P = pathogenic; LP = likely pathogenic; VUS = Variant of unknown significance. *Two of the P variants are present in one patient in compound heterozygosity

Out of 386,353 subjects, screened by enzyme assay, 231,805 adults had at least two ALP measurements, of whom 427 exhibited persistent hypophosphatasaemia (i.e. ≥2 ALP values **≤**35 IU/L and none > 45 IU/L). Thirty-one subjects were excluded because of secondary causes of low ALP levels (detailed in Fig. 1) and 13 because they could not be contacted by telephone. A total of 383 individuals fulfilled the selection criteria and were contacted, of whom 274 declined to participate. Finally, 109 subjects were enrolled and 85 signed the informed consent for the genetic testing. Study overview and selection procedures are depicted in Fig. 1.

The prevalence of persistent hypophosphatasaemia in adults of our clinical setting was 0.18% (see Fig. 1). Genetic test was performed in 85 individuals: 39 (46%) displayed pathogenic (P) or likely pathogenic (LP) variants in *ALPL* (positive genetic test: +GT group), 45 (53%) did not show any P or LP variants (negative genetic test: -GT group) and one subject had a variant of unknown significance (VUS). Segregation analysis in the family of this subject displaying a VUS could not be performed; consequently, this case was excluded from further statistical analysis.

Regarding the subjects who showed variants in *ALPL*, 75% (30/40) were heterozygous for a P variant, 20% (8/40) for a LP variant, 2.5% (1/40) for a VUS, and one patient (2.5%) diagnosed of infantile HPP harbored compound heterozygous pathogenic variants. This patient had a history of multiple recurrent fractures, while subjects displaying variants in heterozygosity exhibited a less severe phenotype. Nine subjects had variants previously associated with odonto-HPP. We identified four variants previously unreported in the literature: two pathogenic variants [c.388_389insG; p.(Val130Glyfs*6) and c.619G > C; p.(Gln207Glu)] and two likely pathogenic variants [c.317A > G; p.(Gln106Arg) and c.547G > A; p.(Asp183Asn)]. The substitutions p.(Gly112Arg), p.(Val128Met), p.(Glu291Lys) and p.(Thr115_Ala116dup) were each present in three subjects; the disease-causing variants p.(Arg152Cys), p.(Asp183Asn), p.(Asp378Gly), p.(Thr166Ile), p.(Gly491Arg) and p.(Glu191Lys) in two subjects and the rest were observed in single subjects. Thirty-four subjects (85%) had missense disease-causing variants, three had duplications (7.5%) and the rest had deletions, insertions or splice site mutations (one subject for each group, respectively). Most of the variants were located in exons 5,6 and 9, were predicted to have a damaging effect in silico pathogenicity prediction tools and were absent or existed at extremely low frequencies in gnomAD. Additional file 1: Table S1 summarizes the complete list of *ALPL* variants and the clinical findings associated to each of them.

Demographic characteristics of study participants are shown in Table 1. Median (IQR) age was 45 (38–55) years in the overall population. Compared with the -GT group, the +GT group showed a lower percentage of females and had a higher body mass index.

**Table 1:** Demographic characteristics of participants, stratified by ALPL genetic test

	+GT^*^ (*N* = 39)	–GT^*^ (*N* = 45)	Total (*N* = 84)	*p* value
Median age (IQR), years	49 (37–63)	44 (38–48)	45 (38–55)	0.092
Female sex, n(%)	23 (59%)	38 (84.4%)	61 (72.6%)	0.009**
Race				
Caucasian,n(%)	37 (94.8%)	45 (100.0%)	82 (97.6%)	0.3
Hispanoamerican, n(%)	1 (2.6%)	0	1 (1.2%)	
Black,n(%)	1 (2.6%)	0	1 (1.2%)	
Median BMI*(IQR), kg/m^2^	24.6 (23.1–28.8)	22.2 (20.4–24.3)	23.5 (21.3–26.6)	0.01**

*GT positive genetic test, *−GT* negative genetic test, *BMI* body mass index. **Significant statistical differences between groups

### Clinical manifestations

Clinical features of +GT and –GT groups are detailed in Table 2. In the univariable analysis, the presence of musculoskeletal pain, premature tooth loss, dental abnormalities, metatarsal stress fractures and history of orthopedic surgery were significantly higher in the +GT group. While metatarsal stress fractures were detected in four subjects in the + GT group, no subject reported this type of fracture in the -GT group.

**Table 2:** Clinical manifestations of participants stratified by *ALPL* genetic testing

Clinical feature, n (%)	+GT* (*N* = 39)	–GT* (*N* = 45)	Total (*N* = 84)	*p* value
Musculoskeletal pain	31 (79.5%)	21 (46.7%)	52 (61.9%)	0.002**
Fractures				
History of fractures	17 (43.6%)	15 (33.3%)	32 (38.1%)	0.334
Multiple fractures	2 (5.0%)	0 (0.0%)	2 (2.4%)	0.129
Peripheral fractures	18 (45.0%)	13 (28.9%)	31 (36.5%)	0.124
Metatarsal fractures	4 (10.0%)	0	4 (4.7%)	0.007**
Family history of fractures	6 (15.8%)	9 (20.0%)	15 (18.1%)	0.619
Orthopedic surgery	7 (18.4%)	2 (4.4%)	9 (10.8%)	0.041**
History of premature teeth loss	6 (15.4%)	1 (2.2%)	7 (8.3%)	0.029**
Dental abnormalities	12 (31.6%)	6 (13.3%)	18 (21.7%)	0.045**
Family history of dental problems	11 (35.5%)	14 (34.1%)	25 (34.7%)	0.906
Muscle weakness	6 (15.4%)	3 (6.7%)	9 (10.7%)	0.198
Calcific periarthritis	4 (10.3%)	3 (6.7%)	7 (8.3%)	0.553
Chondrocalcinosis	2 (5,1%)	0	2 (2,4%)	0.124
Median VAS* (IQR)	3 (2–5)	1 (0–5)	2 (0–5)	0.038**
Median HAQ-DI* (IQR)	0 (0–0.3)	0 (0–0.1)	0 (0–0.3)	0.872

*+ GT positive genetic test, −*GT* negative genetic test, *VAS* Visual Analog Scale, *HAQ-DI* Health Assessment Questionnaire-Disability Index. **Significant statistical differences between groups

Data from the knee and/or pelvis X-ray was available for 11 subjects: 5 in the +GT group and 6 in the -GT group. Among them, two subjects in the former had radiographic chondrocalcinosis signs (one of them with a previous diagnosis of pyrophosphate arthropathy), contrasting with a negative previous history in the -GT group. A higher VAS was observed in the +GT group [3 (2–5) vs 1 (0–5); *p* < 0.05] although functional assessments, as measured by HAQ-DI were comparable in the two genetic groups.

Furthermore, regression models adjusted for possible confounders (age, sex and body mass index) were employed to evaluate the association between clinical manifestations and *ALPL* disease causing variants. The results are shown in Table 3. The only significant association with a positive genetic status was found for the presence of musculoskeletal pain (OR: 7.6; 95% IC: 1.9–30.9). In addition, a trend was also detected for dental abnormalities (OR: 3.6; 95% IC: 0.9–13.4). In this respect, nine subjects carried variants associated with odonto-HPP, of whom only five referred previous dental problems. Metatarsal stress fractures could not be analysed when adjusting for confounders because of convergence problems.

**Table 3:** Results of the logistic regression model adjusted for possible confounders (age, sex and BMI) showing the association between the clinical features and genetic status

Clinical feature	Odds Ratio	95% IC	*p* value
Musculoskeletal pain	7.6	1.9–30.9	0.005**
Peripheral fractures	1.2	0.4–4	0.769
Family history of fractures	0.4	0.06–2.2	0.273
Orthopedic surgery	3.7	0.5–27.1	0.199
History of premature tooth loss	1.7	0.1–21.9	0.673
Dental abnormalities	3.6	0.9–13.4	0.053
Family history of dental problems	0.7	0.2–2.3	0.513
Muscle weakness	3.1	0.4–27.6	0.309
Calcific periarthritis	1.5	0.2–9.9	0.680

**Significant statistical differences between groups

In the +GT group, a median 19-year delay between first signs or symptoms and diagnosis was observed, being the median (IQR) age at the onset of the symptoms of 40.3 years (13.6–50.5) and at the diagnosis of 49.8 (37.2–63.3) years. Median diagnostic delay was 22.7 (19.6–34.6) for those who experienced a pediatric onset (*n* = 9) and 11.9 (7–21.2) for those who did not report symptoms before age 18 (*n* = 29). In terms of initial HPP-associated symptoms, 8 subjects reported dental problems at a median age at onset of 14.51 (7.7–19.5); 18, musculoskeletal symptoms at a median age at onset of 43 (14.8–51.8) and 2 of them, kidney complications.

### Laboratory findings

The biochemical profile of study participants is presented in Table 4. ALP median (IQR) serum levels were significantly lower in the +GT group compared to those in the –GT group [26 IU/L (22–29) vs 29 IU/L (27–32), *p* < 0.005]. In contrast, median phosphate levels were significantly higher in the +GT group compared with the –GT group [4 mg/dL (3.5–4.5) vs 3.4 mg/dL (3.1–4.1), *p* < 0.05, respectively). Eleven percent (5/44) of subjects, based on the available data, presented hyperphosphoremia (> 4.5 mg/dL), most (4/44) in the +GT group. Median calcium levels were within the normal range, statistically comparable in both groups, and hypercalcemia was not observed in subjects using the available data. Levels of 24-h urinary calcium and phosphate excretion did not differ between groups.

**Table 4:** Biochemical variables of study participants, stratified by genetic test results

Biochemical variables, [Median (IQR)]	+GT* (*N* = 39)	–GT* (*N* = 45)	Total (*N* = 84)	*p* value
ALP*, IU/L (*N* = 39, 45, 84)	26 (22–29)	29 (27–32)	28 (25–31)	0.001**
< 20 IU/L	6 (15%)	0 (0%)	6 (7.1%)	
< 25 IU/L	17 (44%)	1 (2%)	18 (21%)	
< 30 IU/L	32 (82%)	25 (56%)	57 (68%)	
< 35 IU/L	38 (97%)	43 (96%)	81 (96%)	
Calcium, mg/dL (*N* = 34, 44, 78)	9.4 (9.1–9.7)	9.2 (9.1–9.4)	9.3 (9.1–9.6)	0.251
Phosphate, mg/dL (*N* = 20,24,44)	4 (3.5–4.5)	3.4 (3.1–4.1)	3.6 (3.2–4.3)	0.016**
Creatinine, mg/dL (*N* = 36, 39,75)	0.91 (0.9–1)	0.9 (0.8–1)	0.9 (0.9–1)	0.053
Urinary calcium excretion, mg/24 h (*N* = 4, 1, 5)	84.5 (22.6–98)	123 (123–123)	89 (41.7–112)	0.400
Urinary phosphate excretion, mg/24 h (*N* = 3, 0, 3)	35 (32.6)	–	35 (32.6)	–

*GT positive genetic test, *−GT* negative genetic test, *ALP* Alkaline phosphatase. **Significant statistical differences between groups

The utility of identifying the presence of *ALPL* variants based on different ALP cut-off levels (20, 25, 30 and 35 IU/L) was assessed (Table 4). The threshold of 25 IU/L served as the best predictor of a positive *ALPL* genetic test in the current study population. Of the 18 subjects with ALP levels below 25 IU/L, 17 had a positive genetic test and only one a negative result. The values for sensitivity, specificity, positive and negative predictive value, and positive and negative likelihood ratio for ALP levels below 25 IU/L were 43.6, 97.8, 94.4, 66.7%, 19.8 and 0.58, respectively (see Table 5).

**Table 5:** Diagnostic utility measures for serum ALP level thresholds

ALP^a^ levels	Sensitivity	Especificity	PPV	NVP	+LR	-LR
< 20 IU/L	15.4%	100%	100%	57.7%	Infinity	0.85
< 25 IU/L	43.6%	97.8%	94.4%	66.7%	19.8	0.58
< 30 IU/L	82.1%	44.4%	56.1%	74.1%	1.48	0.4
< 35 IU/L	97.4%	4.4%	46.9%	66.7%	1.02	0.59

*PPV* positive predictive value, *NPV* negative predictive value, *LR* likelihood ratio, *ALP* Alkaline phosphatase

Of the 65 subjects with ALP levels between 25 and 35 IU/L, we evaluated whether those clinical symptoms potentially related to HPP could improve the detection of a positive variant in our population. In the 36 subjects who presented musculoskeletal pain, 15 (41.7%) displayed a positive variant and 21 (58.3%), a negative genetic test. In addition, 12 subjects reported dental abnormalities (half having an *ALPL* variant) and two individuals diagnosed with HPP experienced metatarsal fractures. The specificity and positive predictive value for musculoskeletal pain in this group of subjects were 52.3 and 41.7%, respectively and 86.4 and 50% for dental abnormalities; thus, the discriminative power did not increase (Additional file 2: Table S2).

## Discussion

Persistently low ALP levels can stem from different etiologies, including HPP. Characterization of the HPP spectrum in adults, which is generally characterized by milder symptoms than in pediatric-onset HPP, is crucial for the correct management and treatment of the disease. In addition, an accurate clinical and biochemical characterization can help distinguish between congenital HPP and secondary hypophosphatasaemia.

In this context, the results of our study are very relevant. Besides establishing the prevalence of an *ALPL* disease-causing variant in adult subjects with persistent hypophosphatasaemia, this is the first study utilizing a common biomarker to identify a positive genetic *ALPL* test.

The prevalence of persistent hypophosphatasaemia in adults of our clinical setting was 0.18%, which is in agreement with two studies, one conducted in a large rural multispecialty clinic population in the USA (prevalence 0.06%) [11] and another in a French tertiary hospital (0.13%) [6]. In our cohort, the subjects included were mainly Caucasians, aged around 50 years and predominantly female, which was congruent with other recent studies [5, 16, 17]. Furthermore, the estimated prevalence of *ALPL* disease-causing variants in these subjects with persistent hypophosphatasaemia was 47%. This means that one out of two subjects with persistent hypophosphatasaemia (secondary causes discarded) had HPP, which is in accordance with the study of Riancho-Zarrabeitia and co-workers [18]. More recently, the study of Mckiernan et al. [19] found a higher proportion of subjects with *ALPL* disease-causing variants (84%), most likely because a more stringent definition of hypophosphatasaemia was used. We identified four previously unreported variants based on the American College of Medical Genetics and Genomics (ACMG) classification criteria [20]. The majority of the variants were missense in nature and were located in exons five, six and nine, as has been commonly observed in public databases and previous reports [9, 18].

According to our results, approximately 50% of persistently low APL levels signify a different underlying etiology than HPP. Therefore, genetic confirmation by *ALPL* genetic testing is required. Nevertheless, in clinical practice, access to such testing is not always available or rapid testing is required. Therefore, it would be very useful to identify the clinical and routine biochemical characteristics of the disease and its predictive value for a proper and early diagnosis, in order to avoid erroneous therapeutic decisions.

Based on the results of this study, neither single nor combined clinical manifestation are useful enough for identifying the presence of an *ALPL* variant. In contrast, ALP levels seem to be discriminative enough for this purpose. ALP levels below 25 IU/L showed a high specificity (97.8%), positive predictive value (94.4%) and positive likelihood ratio (19.8). Consequently, in the presence of symptoms potentially related to HPP and biochemical abnormalities, specific ALP cut-off levels could help diagnostic strategies, especially when genetic testing is not available. If these results are confirmed in other populations, these key findings could prove very useful in clinical practice.

With regards to clinical features, only the presence of musculoskeletal pain was significantly and strongly associated with a positive genetic *ALPL* status. Previous publications also showed a high incidence (41–95%) of musculoskeletal pain in subjects with HPP [5, 15–17]. Indeed, Shapiro et al. recently described musculoskeletal pain attributable to HPP requiring pain medications, such as opioids, as one of the scenarios necessitating treatment with enzyme replacement [21]. However, given the high prevalence of pain in the overall population with hypophosphataseamia, the etiology and characteristics of pain in HPP should be characterized before therapeutic intervention. Metatarsal stress fractures were also more frequent in individuals with *ALPL* disease-causing variants. In a recent publication, metatarsal fractures reached a prevalence of 21% and were considered typical of HPP [16]. The number of subjects presenting this feature was limited and did not allow for any definitive conclusions. Nevertheless, it was the only distinctive clinical symptom related to a positive genetic result when ALP levels were above 25 IU/L.

On the other hand, to implement strategies that will enable an early detection of the disease is mandatory. In our study, a significant 19-year delay was observed, which is in accordance with the global HPP Registry, the largest observational study including real-world data cases [15]. Our study aimed to highlight the distinct clinical characteristics of two groups of subjects with the same biochemical abnormality but with a different genetic status. Clarifying this aspect is an important issue from the diagnostic point of view, given the high clinical variability of the disease [22], the evidence of normal substrates in some adults carrying a defective *ALPL* allele and the limitations of conventional genetic studies [18, 23].

The main weaknesses of our study are the lack of pediatric medical histories, the use of a clinical questionnaire for identifying clinical symptoms and limited data available regarding certain variables, such as radiographic records. As a limitation, the design of the study does not allow us to establish a definitive clinical diagnosis of HPP: although a detailed questionnaire was designed for the evaluation of subjects included, a complete visit and complementary tests targeted to study clinical features or laboratory abnormalities were not performed, so that some subjects with variants in *ALPL* could be carriers. Furthermore, ALP substrates and the analysis of the dominant negative effect of the variants have not been performed due to limitations in their determination.

The main strengths are the thorough analysis of medical records, which yielded a considerably large sample size for a rare disease, the integration of data across a wide range of variables, the analysis of information obtained through a detailed clinical questionnaire and the genetic analysis encompassing this large population. In addition, to the best of our knowledge, this is the first time that ALP cut-off levels are used to identify a positive *ALPL* genetic test, although these results need further investigation.

## Conclusions

In subjects with persistent hypophosphatasaemia, one out of two individuals with primary low ALP levels, presented an *ALPL* disease-causing variant. It is imperative that the clinical and biochemical characteristics and its predictive value are defined if early diagnoses are to be obtained and erroneous therapeutic decisions avoided. Musculoskeletal pain and ALP levels below or equal to 25 IU/L are associated with a positive result in *ALPL* genetic testing. In this scenario, ALP levels below this threshold seem to be very useful for predicting the presence of *ALPL* disease-causing variant. If confirmed in other populations, these key findings can be useful in clinical practice. Prospective studies evaluating these findings and establishing the natural evolution of HPP will definitely enable a comprehensive determination of the disease’s complete clinical spectrum in adults.

## Methods

### Study population and design

This cross-sectional study was performed at La Paz University Hospital (Madrid, Spain). The study adhered to the tenets of the Declaration of Helsinki and approval was obtained from its ethics committee. Each subject provided written informed consent prior to inclusion.

First, 1,536,711 laboratory records including ALP serum levels values from 386,353 subjects were screened to identify abnormally low ALP results. These values were recorded in the biochemical database of the hospital from 2009 to 2015. The main criteria for inclusion were: adults aged 18 years or older with persistent hypophosphatasaemia, defined as ≥2 ALP measurements below or equal to 35 IU/L. Subjects were excluded if ≥1 ALP values were above 45 IU/L (lower limit for adults), or if clinical records revealed secondary underlying causes of hypophosphatasaemia, such as cancer or chemotherapy, adynamic renal osteodystrophy, severe hypothyroidism, Cushing’s disease, Wilson’s disease, nutritional deficiencies, hypomagnesemia, hypozincemia, myocardial infarction, sepsis, major trauma or surgery, massive transfusions and clofibrate therapy among others [11].

### Collected data

Eligible subjects completed a questionnaire to report family or personal history of musculoskeletal pain, muscle weakness, fractures, premature dental loss and previous orthopedic surgery, among other clinical features classically associated with HPP. Additionally, radiographic data were retrieved from medical records. Musculoskeletal pain was considered when symptoms were recurring or chronic (> 6 months) and not when transient and muscle weakness was defined by a chronic subjective perception of a decrease in muscle strength, but no physical examination was performed. Dental abnormalities were defined as tooth shape abnormalities, structure and colour abnormalities of enamel or dentin, thin enamel, late teeth eruption or severe/recurrent cavities and early loss of permanent teeth was defined as the loss of several teeth or extraction (> 10) due to tooth abnormalities in the past, prior to the age of 50. In terms of fractures, peripheral traumatic and fragility ones were evaluated as were stress metatarsal and atypical fractures. Multiple fractures were defined as more than three. Chondrocalcinosis and calcific periarthritis were considered when the subject had a previous diagnosis or if a prior imaging test confirmed the diagnosis. Subjects were required to grade their pain on a 100 mm visual analog scale (VAS) and to complete the Health Assessment Questionnaire-Disability Index (HAQ-DI). Individuals were requested to provide a blood sample for the genetic analysis.

### Laboratory methods

Between 2009 and 2013, the University La Paz Hospital Laboratory utilized an Olympus 5400 analyzer (Beckman Coulter) to measure serum ALP activity. In February 2014, it switched to Siemens Healthineers (Advia 2400 chemistry system) and clinically acceptable correlation and comparison was demonstrated between the two devices (internal Laboratory data available). Both methods measure ALP activity by a kinetic rate method in which p-nitrophenyl phosphate (a colourless organic phosphate ester substrate) is hydrolyzed by ALP to the yellow-coloured product pnitrophenol and phosphate at pH 10,3. Enzymatic activity of ALP is directly proportional to changes in absorbance at 410 nm. The normal adult’s range is 45 to 116 IU/L.

### Genetic analysis

Genomic DNA was extracted from peripheral blood with a Chemagic Blood kit (Perkin Elmer, Waltham, MA) and the screening of the exons and intron/exon boundaries of *ALPL* (NM_000478.4) was performed by Sanger sequencing. In silico pathogenicity prediction and control population frequency analysis were assessed using Alamut V2.6 software (Interactive Biosoftware Rouen, France), Varsome (https://varsome.com/), CADD software (http://cadd.gs.washington.edu) and Silvent et al. criteria [24]. The allelic frequencies were determined using gnomAD (http://gnomad.broadinstitute.org/) and the in silico tools included CADD V1.3, DANN, SIFT, Polyphen, MutationTester, Mutation assessor, FATHM and SpliceSiteFinder-like, MaxEntScan, NNSPLICE, GeneSplicer. The *ALPL* disease-causing variants database (http://www.se-sep.uvsq.fr/03_hypo_mutations.php) was also consulted to obtain up-to-date information about the genetic variants included in our study (Additional file 1: Table S1) already identified [25–36]. Variants were classified according to the American College of Medical Genetics and Genomics (ACMG) standards and guidelines [20].

### Statistical analysis

First, descriptive analysis was employed to determine the prevalence of *ALPL* variants among subjects with persistent hypophosphatasaemia and to compare clinical and laboratory characteristics between individuals with and without *ALPL* variants. Continuous variables were described as median (interquartile range –IQR-) and categorical variables as an absolute number and relative percentage. Comparisons between two independent groups for continuous variables were performed using the Student’s *t*-test for unpaired data if normally distributed, or a Mann-Whitney U test when not. Statistical significance difference between groups for categorical variables was calculated using the Chi-square test or the Fisher’s exact test, as appropriate. Logistic regression models adjusted for confounders were employed to investigate the association between clinical and laboratory characteristics and the genetic status. Finally, diagnostic utility measures (sensitivity, specificity, positive and negative predictive value and positive and negative likelihood ratio) to predict the likelihood of having an *ALPL* variant were calculated. The level of statistical significance was set at *p* < 0.05. Statistical analyses were performed using the IBM SPSS Statistics 23.0 for Windows.

## Supplementary information

**Additional file 1: Table S1.** List of subjects displaying *ALPL* disease causing variants observed in the cohort according to the transcript NM_000478.4, clinical features associated and family history available.

**Additional file 2: Table S2.** Diagnostic utility measures for each of the symptoms combined with ALP levels between 25 and 35 IU/L.

## Data Availability

The datasets generated and/or analyzed during the current study are available from the corresponding author on reasonable request.
