# Supplementary material for: Can we identify individuals with an ALPL variant in adults with persistent hypophosphatasaemia?
Source: Orphanet J Rare Dis. 2020 Feb 17;15:51. doi: 10.1186/s13023-020-1315-y (PMC7026995; doi:10.1186/s13023-020-1315-y)
Supplement: Supplementary file 1 — Additional file 1: Table S1. List of subjects displaying ALPL disease causing variants observed in the cohort according to the transcript NM_000478.4, clinical features associated and family history available. [file 13023_2020_1315_MOESM1_ESM.docx]

**Supplementary table 1.** List of subjects displaying *ALPL* disease causing variants observed in the cohort according to the transcript NM_000478.4, clinical features associated and family history available.

| **ID** | **Sex** | **Age** | **Pain** | **Stress fracture** | **Chondro-calcinosis** | **Prematu-re teeth loss** | **Dental abnor-malities** | **Calcific peri-arthritis** | **Family history of dental problems** | **Family history of fractures** | **Family history + GT*** | **ALP level** | **cDNA change** | **Amino acid** | **ACMG**** | **Exon / Intron** | **Reference** |
| --- | --- | --- | --- | --- | --- | --- | --- | --- | --- | --- | --- | --- | --- | --- | --- | --- | --- |
| 1 | M | 54 | Yes | No | No | No | No | Yes | Yes | No | No | 25 | c.871G>A | p.Glu291Lys | P | Exon 9 | Mornet et al., 1998 (25) |
| 2 | M | 63 | Yes | No | Yes | Yes | No | No | No | No | No | 18 | c.334G>C | p.Gly112Arg | P | Exon 5 | Whyte et al., 2015 (26) |
| 3 | M | 75 | Yes | No | No | No | No | No | No | No | Yes | 19 | c.497C>T | p.Thr166Ile | P | Exon 8 | Riancho-Zarrabeitia et al., 2016 (18) |
| 4 | M | 63 | Yes | No | No | No | No | Yes | No | No | No | 29 | c.871G>A | p. Glu291Lys | P | Exon 9 | Mornet et al., 1998 (25) |
| 5 | F | 42 | No | Yes | No | No | No | No | No | Yes | Yes | 22 | c.871G>A | p.Glu291Lys | P | Exon 9 | Mornet et al., 1998 (25) |
| 6 | M | 44 | Yes | No | No | No | No | No | No | No | No | 15 | c.1366G>A | p.Gly456Arg | P | Exon 12 | Ozono et al., 1996 (27) |
| 7 | F | 32 | Yes | Yes | Yes | No | No | No | Yes | Yes | Yes | 20 | c.343_348dup | p.Thr115_Ala116dup | P | Exon 5 | Versailles lab, 2009 |
| 8 | M | 65 | Yes | No | No | Yes | No | No | No | No | No | 30 | c.334G>C | p.Gly112Arg | P | Exon 5 | Whyte et al., 2015 (26) |
| 9 | M | 76 | Yes | No | No | Yes | Yes | No | No | No | No | 24 | c.1022A>G | p.His341Arg | P | Exon 10 | Versailles lab, 2018 |
| 10 | F | 37 | Yes | Yes | No | No | No | No | Yes | No | No | 29 | c.382G>A | p.Val128Met | P | Exon 5 | Mumm et al., 2002 (28) |
| 11 | M | 68 | Yes | No | No | No | No | No | No | Yes | No | 35 | c.382G>A | p.Val128Met | P | Exon 5 | Mumm et al., 2002 (28) |
| 12 | F | 42 | Yes | No | No | No | No | No | Yes | No | No | 24 | c.454C>T | p.Arg152Cys | P | Exon 5 | Versailles lab, 2009 |
| 13 | M | 30 | No | No | No | No | No | No | Yes | Yes | Yes | 33 | c.1133A>G | p.Asp378Gly | P | Exon 10 | Tenorio et al., 2017 (29) |
| 14 | F | 36 | No | No | No | No | No | No | No | No | No | 25 | c.317A>G | p.Gln106Arg | LP | **Exon 5** | **This study** |
| 15 | F | 35 | Yes | No | No | No | Yes | No | No | No | Yes | 20 | c.659G>T | p.Gly220Val | P | Exon 12 | Taillandier et al., 2001 (30) |
| 16 | V | 60 | Yes | No | No | No | No | No | No | No | No | 29 | c.1471G>A | p.Gly491Arg | LP | Exon 12 | Mornet et al., 1998 (25) |
| 17 | F | 62 | Yes | No | No | No | No | No | Yes | No | Yes | 23 | c.1283G>A | p.Arg428Gln | P | Exon 11 | Versailles lab, 2010 |
| 18 | F | 57 | Yes | No | No | No | Yes | Yes | Yes | Yes | Yes | 21 | c.343_348dup | p.Thr115_Ala116dup | P | Exon 5 | Versailles lab, 2009 |
| 19 | F | 49 | Yes | No | No | No | No | No | No | No | No | 23 | c.473-2A>G | c.473-2A>G | P | Intron 5 | Riancho-Zarrabeitia et al.,2016 (18) |
| 20 | F | 49 | Yes | No | No | No | No | No | No | No | No | 23 | c.892G>A | p.Glu298Lys | P | Exon 9 | Orimo et al., 1994 (31) |
| 21 | M | 72 | Yes | No | No | No | No | No | No | No | No | 27 | c.343_348dup | p.Thr115_Ala116dup | P | Exon 5 | Versailles lab, 2009 |
| 22 | F | 43 | No | No | No | No | No | No | No | No | No | 29 | c.1348C>T | p.Arg450Cys | P | Exon 12 | Mornet et al., 1998 (25) |
| 23 | F | 55 | Yes | No | No | Yes | Yes | No | Yes | No | Yes | 31 | c.809G>A | p.Trp270* | P | Exon 8 | Mornet et al., 1998 (25) |
| 24 | M | 46 | No | No | No | No | Yes | No | No | No | No | 28 | C.659G>C | p.Gly220Ala | LP | Exon 6 | Spentchian et al., 2003 (32) |
| 25 | F | 25 | Yes | No | No | No | Yes | No | No | No | No | 26 | c.454C>T | p.Arg152Cys | P | Exon 5 | Versailles lab, 2009 |
| 26 | M | 41 | No | No | No | No | No | No | No | No | Yes | 27 | c.497C>T | p.Thr166Ile | LP | Exon 6 | Riancho-Zarrabeitia et al., 2016 (18) |
| 27 | F | 74 | Yes | No | No | Yes | Yes | Yes | No | No | No | 23 | c.984_986del | p.Phe328del | VUS | Exon 9 | Orimo et al., 1997 (33) |
| 28 | F | 22 | Yes | No | No | No | No | No | No | No | No | 32 | c.871G>T | p.Glu291* | P | Exon 9 | Taillandier et al., 2000 (34) |
| 29 | F | 36 | Yes | No | No | No | Yes | No | Yes | No | No | 34 | c.571G>A | p.Glu191Lys | P | Exon 6 | Henthorn et al., 1992 (35) |
| 30 | F | 33 | Yes | No | No | No | Yes | No | No | No | No | 22 | c.1471G>A | p.Gly491Arg | LP | Exon 12 | Mornet et al., 1998 (25) |
| 31 | F | 67 | Yes | No | No | Yes | Yes | No | Yes | No | No | 28 | c.388_389insG | p.Val130Glyfs*6 | P | **Exon 5** | **This study** |
| 32 | F | 54 | Yes | No | No | No | No | No | No | No | No | 13 | c.382G>A | p.Val128Met | P | Exon 5 | Mumm et al., 2002 (28) |
| 33 | F | 40 | Yes | No | No | No | Yes | No | No | No | No | 19 | c.334G>C | p.Gly112Arg | P | Exon 5 | Whyte et al., 2015 (26) |
| 34 | F | 23 | No | No | No | No | No | No | No | No | Yes | 31 | c.547G>A | p.Asp183Asn | LP | **Exon 6** | **This study** |
| 35 | F | 54 | Yes | No | No | No | No | Yes | Yes | No | No | 22 | c.551G>A | p.Arg184Gln | P | Exon 5 | Versailles lab. Nov 2014 |
| 36 | F | 63 | Yes | No | No | No | No | No | No | No | No | 27 | c.547G>A | p.Asp183Asn | LP | **Exon 6** | **This study** |
| 37 | M | 55 | No | No | No | No | No | No | No | No | No | 26 | c.619G>C | p.Gln207Glu | P | **Exon 6** | **This study** |
| 38 | M | 72 | Yes | No | No | No | Yes | No | No | No | Yes | 28 | c.1426G>A | p.Gly476Lys | LP | Exon 5 | Taillandier et al., 1999 (36) |
| 39 | M | 44 | Yes | No | No | No | No | No | No | No | No | 28 | c.1277G>A | p.Gly426Asp | P | Exon 11 | Mumm et al., 2002 (28) |
| 40 | F | 56 | Yes | Yes | No | Yes | Yes | No | No | Yes | Yes | 11 | c.(571G>;(1133A>G) | p.[Glu191Lys]  [Asp378Gly] | P  P | Exon 6  Exon10 | Henthorn et al., 1992 (35)  Tenorio et al., 2017 (29) |

* GT= positive genetic test; ** ACMG = American College of Medical Genetics and Genomics.
