# Supplementary material for: Can we identify individuals with an ALPL variant in adults with persistent hypophosphatasaemia?
Source: Orphanet J Rare Dis. 2020 Feb 17;15:51. doi: 10.1186/s13023-020-1315-y (PMC7026995; doi:10.1186/s13023-020-1315-y)
Supplement: Supplementary file 2 — Additional file 2: Table S2. Diagnostic utility measures for each of the symptoms combined with ALP levels between 25 and 35 IU/L. [file 13023_2020_1315_MOESM2_ESM.docx]

**Table S2.** Diagnostic utility measures for each of the symptoms combined with ALP levels between 25-35 IU/L.

| **ALP levels (IU/L)** | **S** | **E** | **PPV** | **NVP** | **+ LR** | **- LR** |
| --- | --- | --- | --- | --- | --- | --- |
| **Musculoskeletal pain** | 71.4% | 52.3% | 41.7% | 79.3% | 1.5 | 0.55 |
| **Dental abnormalities** | 28.6% | 86.4% | 50% | 71.7% | 2.1 | 0.83 |
| **Orthopedic surgery** | 19% | 95.5% | 66.7% | 71.2% | 4.22 | 0.85 |
| **Both musculoskeletal pain and dental abnormalities** | 50% | 87% | 62.5% | 80% | 3.85 | 0.57 |

S = sensitivity; E = specificity, PPV = positive predictive value; NPV = negative predictive value; + LR = positive likelihood ratio; - LR = negative likelihood ratio
